# FeHf Binary Hydroxide/Oxide Nanostructures as Catalysts for Oxygen Evolution

**DOI:** 10.1021/acsanm.5c00912

**Published:** 2025-04-19

**Authors:** Biswaranjan D. Mohapatra, Mateusz Szczerba, Joanna Czopor, Daniel Piecha, Marcin Pisarek, Grzegorz D. Sulka

**Affiliations:** † Department of Physical Chemistry and Electrochemistry, Faculty of Chemistry, Jagiellonian University, Gronostajowa 2, 30-387 Krakow, Poland; ‡ Doctoral School of Exact and Natural Sciences, Jagiellonian University, Lojasiewicza 11, 30-348 Krakow, Poland; § Laboratory of Surface Analysis, Institute of Physical Chemistry, Polish Academy of Sciences, Kasprzaka 44/52, 01-224 Warsaw, Poland

**Keywords:** electrodeposition, nanocomposites, FeHf binary hydroxide/oxide, electrocatalysis, oxygen evolution reaction

## Abstract

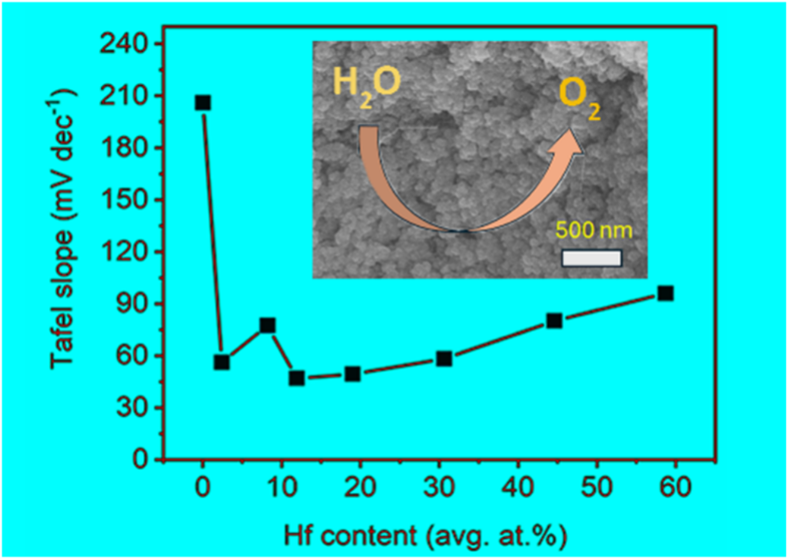

We present pulsed electrodeposition (PED) of FeHf binary hydroxide/oxide (FeHf-BH) nanocomposites from aqueous electrolyte baths containing NO_3_^–^ ions. The deposition was carried out on a graphite foil at room temperature. This study, for the first time, demonstrated a controlled variation of Fe (5.9–49.9 avg. at. %) and Hf (2.4–58.7 avg. at. %) in the deposited materials. We showed the high scalability of FeHf-BH deposition by tuning the PED parameters. The morphology, composition, chemical structure, and oxidation states of metals in the materials were investigated by using scanning electron microscopy (SEM), energy dispersive X-ray spectroscopy (EDS), X-ray diffraction (XRD), Raman spectroscopy, and X-ray photoelectron spectroscopy (XPS). The deposited materials consist of agglomerated nanoparticles sized 50–150 nm. Thermal annealing studies revealed improved crystallinity, with the appearance of thermodynamically stable oxide phases of Fe_3_O_4_, Fe_2_O_3_, and HfO_2_ in the composites. The oxygen evolution activity of the materials was analyzed in an alkaline medium based on the Hf content. The optimized material containing 11.9 avg. at. % Hf demonstrated an OER onset potential of 1.63 V vs RHE, Tafel slope of 47 mV dec^–1^, and required only 470 mV overpotential to reach a 50 mA cm^–2^ OER current. These PED strategies of designing FeHf-BH materials may open an avenue for designing other catalytically active and stable multimetallic hydroxides/oxides composites.

## 1 Introduction

Multimetallic oxide/hydroxide nanocomposites containing valve metals (e.g., W, Ti, Ta, or Hf) and iron group metals (Fe, Co, and Ni) are considered one of the most important classes of materials due to their diverse applications, including electronic devices, photocatalysis, photoelectrocatalysis, and biomaterials.^1−7^ However, the synthetic procedures for obtaining these nanocomposites often involve costly and time-consuming methods, such as vacuum sputtering under high-temperature conditions, chemical vapor deposition (CVD), atomic layer deposition (ALD), solvothermal synthesis, and sol–gel-based spin coating.^7−12^ Among various electrochemical approaches, such as anodic oxidation and electrodeposition, cathodic electrodeposition of transition metal oxides/hydroxides from aqueous/nonaqueous solutions offers several advantages over other synthetic methods.^13−15^ These advantages include the use of low cost equipment, the ability to cover large deposition areas, high scalability, low operating temperature, and the gentle processing of materials.^13−22^ Moreover, among the various types of aqueous electrodeposition baths, those containing chloride and/or nitrate ions are considered the most favorable for electrodeposition of oxides or hydroxides of first-row transition metals (Fe, Co, Ni, and Zn).^16−22^ In these cases, it is understood that the electrochemical reduction of nitrates (eq 1) and/or the reduction of water (eq 2) generates necessary hydroxide anions at the electrode–electrolyte interface for the deposition (eq 3) of the corresponding metal oxide/hydroxides.^13,14^


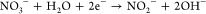
1
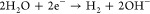
2
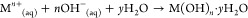
3where M is the transition metal.

Over the past decade, some studies have suggested that the electrodeposition of binary and ternary metal oxide/hydroxide of first-row transition metals can also be successfully performed from chloride and/or nitrate ions baths.^17−21^ For example, Zeng et al. reported a flower-like structure of binary Co_3_O_4_–NiO by cathodic electrodeposition from NO_3_^–^-containing bath at −1.0 V vs Ag/AgCl.^18^ Ye and Yan demonstrated the cathodic deposition of NiFe (oxy)hydroxide and NiCeO_*x*_H_*y*_ from aqueous baths containing NO_3_^–^.^19,20^ Fujita et al. reported the cathodic deposition of Fe–Ce–O films from an aqueous bath containing chloride and sulfate ions.^22^ In this case, the cathodic reduction of dissolved oxygen (O_2_ + 2H_2_O + 2e^–^ → H_2_O_2_ + 2OH^–^) was considered to be a potential source of OH^–^ ions for the deposition of binary metal oxides. Recently, Abebe et al. showed the electrodeposition of ternary metal (Ni, Co, and Mn) oxides from nitrate baths at −0.9 V vs Ag/AgCl.^21^ In this study, it was demonstrated that, regardless of the change in molar ratios of metal ions in the electrolyte, the amount of cobalt was found to be the highest and Mn the lowest in the deposited ternary metal oxides. Despite these advancements, the conditions for the cathodic deposition and codeposition of valve metal-based oxides/hydroxides or its composites with other transition metals remain unexplored in aqueous media, particularly when compared to iron-group metal oxides/hydroxides. Further studies in this area are still needed.

However, it is well-known that compared to constant potential deposition (CPD), pulsed electrochemical deposition (PED) offers a better control over the properties of deposited materials due to multiple adjustable electrochemical parameters.^14,23,24^ The high number of variables, such as cathodic (*V*_on_)/anodic (*V*_off_) pulses, pulse time (*t*_on_/*t*_off_), or duty cycle in the PED setups, can effectively regulate the transport of metal ions and their concentration at the interface.^23,24^ As a result, PED provides superior control over the macroscopic properties, composition, and uniformity of deposited materials.^23,24^ In contrast, continuous deposition often leads to inhomogeneous growth of nuclei, which can have detrimental effects on coating properties.^13,14^ Although the effects of various PED parameters on the physicochemical properties of deposited iron oxides/hydroxides have been well studied, the conditions for codeposition of binary M-Fe oxides/hydroxides (where M represents valve metals) with tunable M:Fe ratios have not yet been fully explored.

In this study, we employed PED setups for coelectrodeposition of FeHf hydroxide/oxides (HfFe-BHs) from aqueous solutions. We investigated the influence of various PED parameters (e.g., pulse on/off potential, pulse on/off time, and total cycle time) as well as the concentrations of Hf and Fe ions on the morphology, composition, and weight of the deposited materials. We demonstrated the ability to control the stoichiometric ratio of Hf:Fe in the deposited materials by adjusting the concentration ratios of Hf to Fe ions in the deposition bath. Furthermore, we evaluated the electrocatalytic oxygen evolution activity of the deposited materials in alkaline media, examining the effects of Hf-to-Fe content variation on the OER performance. Our results showed that an optimal Hf content is beneficial for the OER activity.

## 2 Experimental Section

### 2.1 Materials and Chemical Reagents

KCl (99.5%, Chempur), KF (≥99.5%, Sigma-Aldrich), KNO_3_ (≥99%, Sigma-Aldrich, ACS reagent), FeCl_3_·6H_2_O (97%, Sigma-Aldrich, ACS reagent), HfCl_4_ (98%, Sigma-Aldrich), KOH (85.5%, Chempur), RuO_2_ (99.9%, Sigma-Aldrich), acetone (Stanlab), and Vulcan XC72 conductive carbon black (Nanografi) were used as received. A graphite foil (99.8%, thickness of 0.4 mm) was purchased from Thermo-Scientific. The water used throughout the experiments was purified with a Milli-Q system from Millipore (resistivity of 18 MΩ cm).

## 2.2 Electrochemical Deposition

Before electrodeposition, the graphite foil was cut into 2 cm × 1 cm coupons and cleaned with acetone followed by ethanol for 1–2 min using ultrasonication. Then, the working area was properly limited by coating a part of the graphite with an acid-resistant paint (Protecting Lacquer Yellow, Enthone GmbH). Electrodeposition was carried out by using a PGSTAT 204 potentiostat/galvanostat connected to a three-electrode cell. The graphite foil (exposed area of 1 cm × 1 cm) served as the working electrode, a Pt mesh (2 cm × 3 cm) acted as the counter electrode, and a saturated calomel electrode (SCE) was used as the reference electrode. The electrodeposition bath consisted of aqueous solutions containing KCl (1 M), KNO_3_ (0.5 M), and various concentrations of FeCl_3_ and HfCl_4_. Table S1 shows the choice of concentrations of FeCl_3_ and HfCl_4_ and their molar ratios in the electrolytes. HfFe binary hydroxides were deposited onto the graphite foil by using PED with varying *V*_on_ potentials (−1.6, −1.7, or −1.8 V) and *t*_on_ times (0.1, 0.2, 0.35, or 0.5 s), while maintaining a constant *V*_off_ potential of −0.5 V and a *t*_off_ time of 1 s. The total duration of PED varied from 2 to 60 min. To avoid oxygen reduction during deposition, the solution was deaerated with nitrogen gas, and a nitrogen atmosphere was maintained above the solutions during the deposition process. All electrodeposition experiments were conducted in an unstirred solution at room temperature. After electrodeposition, the fabricated graphite foils were removed from the deposition bath and gently rinsed with deionized water to remove excess electrolyte ions from the surface of the deposited materials. Afterward, the samples were dried under a hot air flow and stored in sample vials for further analysis and application. The acid-resistant coatings were removed gently before the analysis of the samples. Selected specimens were thermally treated in a muffle furnace (FCF 5SHM Z, Czylok) at 500 °C for 2 h in an air atmosphere, with a heating rate of 2 °C min^–1^.

### 2.3 Materials Characterization

The morphological and elemental characterization of the deposited materials was performed by using field emission scanning electron microscopy (FE-SEM/EDS, Hitachi S-4700 with a Noran System 7, Japan) operated at 20 kV. Before FE-SEM/EDS analysis, the samples were sputtered with gold to enhance their conductivity. X-ray diffraction (XRD) measurements for the samples were performed at operating voltage of 40 kV and 15 mA current by a Malvern Panalytical Aeris diffractometer. Cu Kα radiation (1.54 Å) was used as X-ray source and the data were recorded at 2θ range of 10–90° with a step size of 0.04° and a scan rate of 5.55° min^–1^. Raman measurements of the materials were performed at room temperature using a Horiba XploRA PLUS motorized confocal Raman microscope, equipped with a 532 nm excitation laser and a nominal power of 100 mW. The laser was focused on the sample with a 50× objective (Olympus LMPlanFL N, *N*_A_ = 0.5), and the laser power filter was set to 1%. To achieve a high signal-to-noise ratio, an 1800 gr mm^–1^ grating and a pinhole slit width of 300 μm were used. The acquisition time was set to 2 s with 10 accumulations per spectrum. An intensity correction system (ICS) was used during the recording of the spectra. The surface elemental composition and oxidation states of the elements were analyzed using an X-ray photoelectron spectrophotometer (PHI 5000 VersaProbe, ULVAC-PHI, 2500 Hagisono, Chigasaki, Kanagawa, Japan) with monochromatic Al Kα radiation (*h*ν = 1486.6 eV). The X-ray source operated with a 100 μm spot size at 25 W and 15 kV. The analyzed area was a 500 μm square. High-resolution XPS spectra were collected using a hemispherical analyzer at a pass energy of 23.5 eV with an energy step of 0.1 eV. The X-ray beam was incident at an angle of 45° relative to the surface normal, and the analyzer axis was positioned at a 45° angle with respect to the surface. XPS data were processed using Avantage software (ver. 5.9911 Thermo Fisher Scientific). Deconvolution of the XPS spectra was performed by using a Smart background and a Gaussian peak shape with 35% Lorentzian character. The binding energy (BE) scale was referenced to the C 1s peak at a BE = 284.0 eV.

### 2.4 Electrochemical Measurements

The electrochemical activity toward the oxygen evolution reaction of the deposited materials was studied at room temperature using a three-electrode cell containing 1 M KOH aqueous electrolyte (pH = 14). The electrodeposited materials served as the working electrode, a Pt mesh acted as the counter electrode, and a saturated calomel electrode (SCE) was used as the reference electrode. The electrodes were connected to an electrochemical workstation (PGSTAT 204, Autolab or VMP-300, BioLogic potentiostat/galvanostat) and the OER catalytic activity was evaluated by linear sweep voltammetry (LSV) from 0.2 to 0.8 V vs SCE at a scan rate of 10 mV s^–1^. For comparison, a graphite foil coated with 20 wt % RuO_2_ was tested under similar conditions. The catalytic stability of the materials was assessed by using chronopotentiometry at a current density of 10 mA cm^–2^ for about 8000 s. To compare the catalytic activity of our materials with other reported non-noble metal-based oxide/hydroxides, the applied potentials in this study were converted to reversible hydrogen electrode (RHE) by applying Nernst equation; *E* (V vs RHE) = *E* (V vs SCE) + 0.059pH + 0.244, where 0.244 V is the standard potential for the SCE electrode at 25 °C.^12^

## 3 Results and Discussion

**Figure 1:**
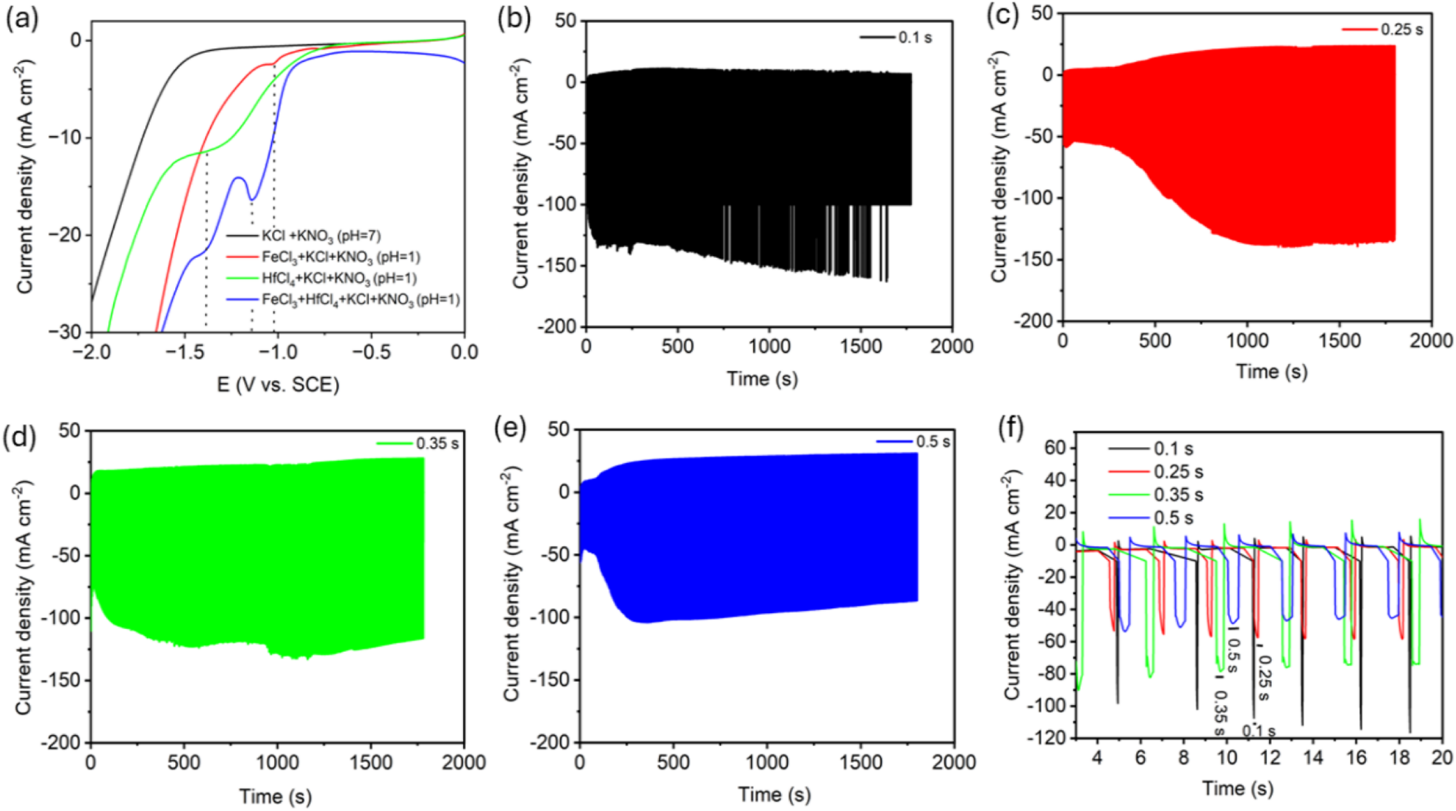
(a) Cathodic polarization curves on graphite foil electrodes in four different nitrate solution baths; The concentration of KCl, KNO_3_, Fe, and Hf precursors are 1 M, 0.5 M, 30 mM, and 50 mM respectively. Current density–time curves of FeHf-BH PED at −1.8 V (*V*_on_) using electrolytes containing 30 mM Fe^3+^ + 50 mM Hf^4+^ ions with varying duration of *t*_on_= (b) 0.1, (c) 0.25, (d) 0.35, and (e) 0.5 s. (f) Zoomed-in view of the current density–time curves from panel (b–e).

**Scheme 1:**
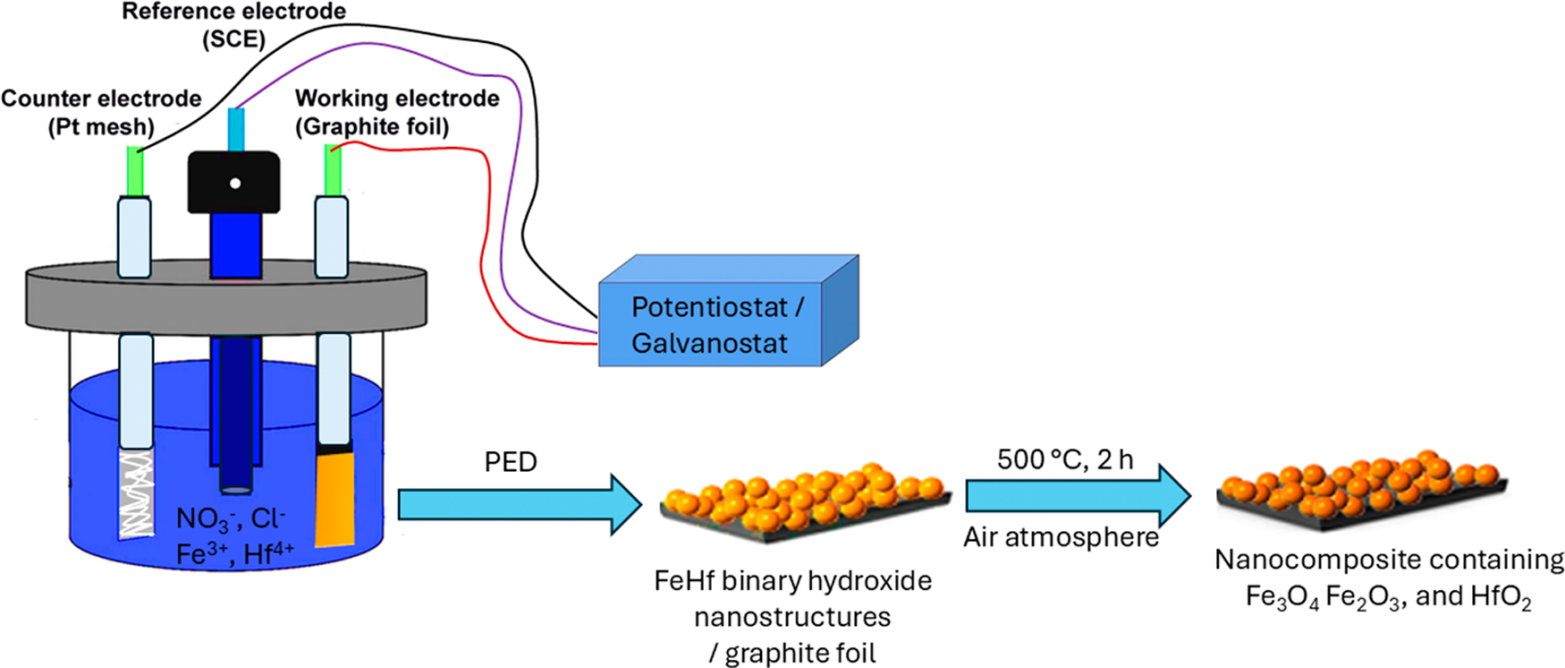
A Pictorial Representation Showing Arrangement of Electrodes for PED Experiment and the Thermal Treatment Conditions for the Deposited Materials

Scheme 1 shows the pictorial representation of the arrangement of electrodes and post treatment conditions for the PED deposited materials. Prior to performing PED of FeHf-BH materials, the individual precursor solutions were characterized by cathodic scanning from 0 to −2.0 V versus SCE at 10 mV s^–1^ (Figure 1a). For all of the samples, the current remained nearly zero and no redox behavior was observed from 0.0 to −0.75 V. In the solution without transition metal ions, no reduction peaks were observed until −1.4 V. A sharp increase in reduction current below −1.4 V likely indicates both nitrate and water reduction processes (eqs 1 and 2).^14,24^ However, compared to the KCl + KNO_3_ solution, the polarization curves in solutions containing 30 mM Fe^3+^ ions, 50 mM Hf^4+^ ions, and a mixture of 30 mM Fe^3+^ + 50 mM Hf^4+^ ions exhibited an early increase in reduction currents with higher current values in the potential range of −1.4 to −2.0 V. Many previous studies have associated these behavior with the reduction of nitrate ions induced by the transition metal ions.^15,21^ Notably, in case of tantalum oxide deposition, H. Hajibabaei et al. demonstrated that the presence of KCl in the electrolyte can significantly shift the onset potential for the reduction current and can also cause an increase in cathodic current.^15^ This behavior could be attributed to improved ionic conductivity due to the introduction of K^+^ and Cl^–^ ions into the electrolyte bath.

In our case, we observed cathodic peaks appearing just before the sharp increase in the water reduction current, which suggests the reduction of metal ions. The reduction peak at about −1.03 V for Fe^3+^ containing electrolytes and at −1.35 V for Hf^4+^ containing electrolytes indicates reduction of the respective metal ion species to their lower oxidation states. Notably, two reduction peaks at about −1.15 and −1.35 V were observed for the solution containing both Fe^3+^ and Hf^4+^ ions, indicating the reduction of metal ion species. In a previous study on electrodeposition of Fe oxides, S. Perez-Villar et al. reported a reduction peak at about −1.5 V, attributing it to the stepwise reduction of Fe(III) to Fe(0) via Fe(II).^24^ For all of the electrolytes containing nitrate, the reduction of NO_3_^–^ and H_2_O can also be considered for local change in pH. Interestingly, a pale yellow deposit was observed only for the solution containing both Fe^3+^ and Hf^4+^ ions. The deposition from the bath in the presence of only Fe^3+^ ions resulted in an unstable Fe-hydroxide/oxide layer that easily detached when the electrode was removed from the electrodeposition bath. No significant deposition or changes in substrate weight were observed in baths containing only Fe^3+^ or Hf^4+^ ions, indicating unsuitable conditions for the individual deposition of these metal hydroxides/oxides. However, codeposition of FeHf hydroxides/oxides was successfully achieved from the solution containing both Fe^3+^ and Hf^4+^ ions. Based on these findings, PED parameters were optimized, with the deposition potential (*V*_on_) set to −1.4 V or lower (i.e., −1.6, −1.7, and −1.8 V), and the pulse duration (*t*_on_) varied between 0.1, 0.25, 0.35, and 0.5 s. The pulse off time (*t*_off_) was set to 1 s, and the total PED duration was adjusted from 2 to 60 min to evaluate the effects of these parameters on the surface morphology, composition, and uniformity of the deposited materials.

Figure 1b-e shows the current–time curves for the PED of FeHf-BH at −1.8 V with different durations of *t*_on_ (0.1, 0.25, 0.35, and 0.5 s). The result indicates that for all *t*_on_ variations, the maximum PED current ranged between −45 and −180 mA cm^–2^. Figure 1f shows the typical pulse potential waveform of PED during the first 20 s for four different durations of *t*_on_. As can be seen, an increase in current during the pulse-on period indicates the nitrate ion reduction and hydrogen evolution reactions, which generate the necessary base ions for the deposition of FeHf-BH. During the t_off_ period (1.0 s), electrolyte ions can migrate to the depleted area at the electrode–electrolyte interface, while during the *t*_on_ period, the metal ions are available for nucleation and growth of the hydroxide/oxide crystals. The number of crystal grains per unit area increases with the number of pulse cycles, causing the electrodeposited material to continue growing on the substrate. Figure S1 (Supporting Information) shows photographic images of the deposited materials with different *t*_on_ durations, i.e., 0.1, 0.25, 0.35, and 0.5 s, indicating that the coatings are uniform for up to *t*_on_ of 0.35 s. In contrast, the coating becomes nonuniform at the highest *t*_on_ value of 0.5 s, indicating that a controlled pulse-on duration is crucial for achieving uniformity in the deposited film. Moreover, variation in the pulse potential studies for this electrolyte revealed no material deposition above −1.6 V (*V*_on_). Significant and uniform deposition was observed at *V*_on_ values of −1.7 and −1.8 V (Figure S2, Supporting Information). Therefore, based on these analyzes, we selected *V*_on_ of −1.8 V and *t*_on_ of 0.35 s for further studies in this system.

**Figure 2:**
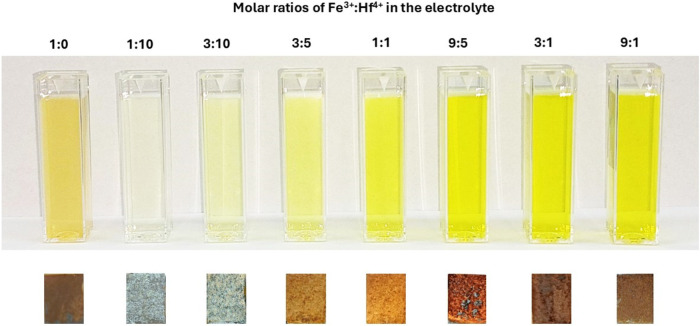
Digital photographs of the electrolyte solutions and the corresponding electrodeposited Fe–H and FeHf-BH layers on the graphite substrate. Each electrodeposition was carried out by applying −1.8 V (*V*_on_), 0.35 s (*t*_on_), −0.5 V (*V*_off_), and 1.0 s (*t*_off_) for 30 min.

**Figure 3:**
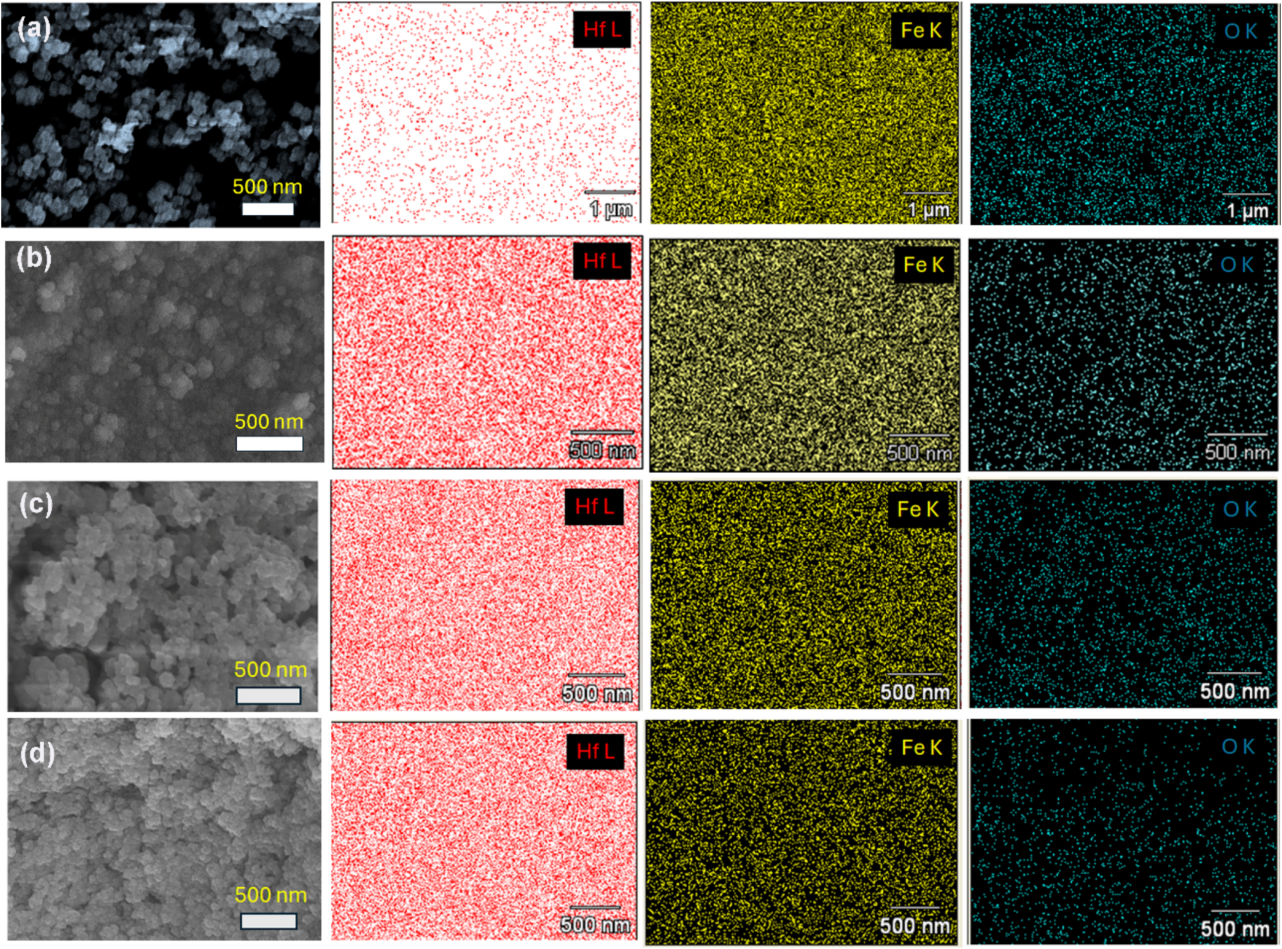
FE-SEM images and corresponding EDS elemental mapping images of Hf, Fe and O for the FeHf-BH materials electrodeposited from the electrolyte containing (a) 9:1, (b) 1:1, (c) 3:10, and (d) 1:10 molar ratios of Fe^3+^/Hf^4+^. Samples were obtained at −1.8 V (*V*_on_), 0.35 s (*t*_on_), −0.5 V (*V*_off_), and 1.0 s (*t*_off_) for 30 min. Elemental mapping images of Hf, Fe, and O are displayed horizontally on the right of the FE-SEM images.

Figure 2 shows a series of digital photos of electrolyte solutions with variations in the Fe^3+^ to Hf^4+^ ions ratio, along with the corresponding samples electrodeposited on the graphite substrate through PED at −1.8 V (*V*_on_) and 0.35 s (*t*_on_) for 30 min. All of the nitrate-ion-based electrolytes containing Fe^3+^ and Hf^4+^ ions were clear, transparent, and stable for up to 4 weeks after preparation. This indicates that no metal hydrolysis or precipitation occurred in the electrolytes. Figure 3a-d displays the FE-SEM images of the FeHf-BH materials electrodeposited from the electrolytes containing various concentrations of Fe^3+^ and Hf^4+^ ions along with the corresponding EDS elemental mapping images positioned horizontally to the right of the FE-SEM images. As observed, regardless of the variations in the concentrations of Fe^3+^ and Hf^4+^ ions, the morphology of the obtained FeHf-BH materials is consistent, featuring agglomerated spherical particles with a size of about 50–150 nm. These results indicate no significant change in the structural morphology of the electrodeposited materials with varying concentrations of Fe^3+^ and Hf^4+^ ions.

**Figure 4:**
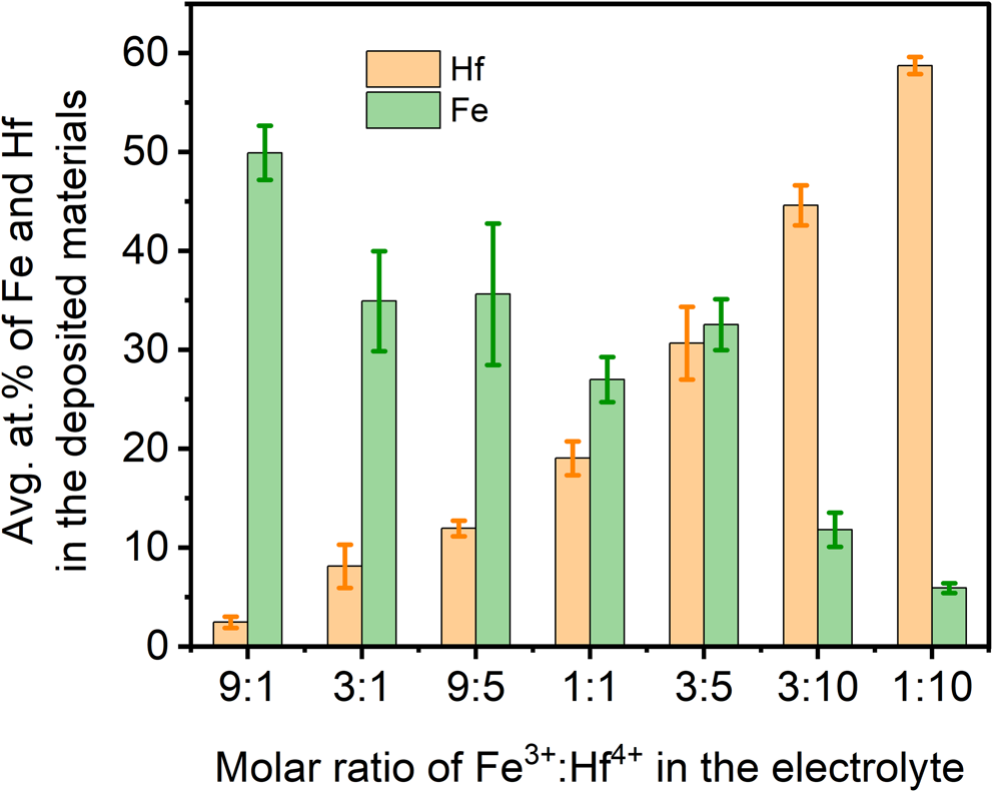
Average at. % of Fe and Hf in electrodeposited FeHf-BH nanostructures obtained at PED conditions of −1.8 V (*V*_on_), 0.35 s (*t*_on_), −0.5 V (*V*_off_), and 1.0 s (*t*_off_) for 30 min.

The elemental mapping images for Hf, Fe, and O for the FeHf-BH nanostructures presented in Figure 3a-d show a uniform distribution of Hf, Fe, and O throughout the materials. However, the EDS mapping clearly shows a decrease in the density of Fe spots and an increase in the density of Hf spots in the deposited materials as the Fe^3+^/Hf^4+^ ratio in the electrolyte decreases. To further confirm these observations, the average atom % of Hf and Fe in electrodeposited FeHf binary hydroxides were analyzed and tabulated in Table S2 (Supporting Information). Figure 4 presents a histogram of Fe and Hf contents in the obtained materials as the Fe^3+^/Hf^4+^ molar ratio in the electrolytes changes. The error bars in the plot indicate the standard deviation values for the metal contents (atom %) in the composites. As the Hf^4+^ content increases in the electrolyte, a gradual increase in the at. % of Hf in the films is observed. Similar observations of increasing the at. % of Fe are also noted. This result suggests that the Hf and Fe contents in the deposited FeHf-BH layers can be controlled by adjusting the Fe^3+^/Hf^4+^ ratio in the electrolyte. For example, a maximum average atomic percentage of 58.7% Hf with 5.9% Fe in the deposited FeHf-BH layer was obtained by using an electrolyte containing 1:10 molar ratio of Fe^3+^/Hf^4+^ (Figure 4). For simplicity, the sample was named according to the Hf content in the material, i.e., FeHf-BH-58.7. Similarly, the other electrodeposited materials are designated as FeHf-BH-44.6, FeHf-BH-30.6, FeHf-BH-19, FeHf-BH-11.9, FeHf-BH-8.2, and FeHf-BH-2.4 representing average atomic percentages of Hf equal to 44.6, 30.6, 19, 11.1, 8.2, and 2.4, respectively. Furthermore, the total charge involved in the PED experiments were calculated and correlated to the Fe and Hf contents in the deposited materials (Figure S3, Supporting Information). It is found that the charge involved in the deposition of FeHf-BH-58.7 (10.53 Q cm^–2^) and FeHf-BH-44.6 (16.36 Q cm^–2^) samples is significantly lower compared to that of the other materials, which may be due to the lower concentration (≤15 mM) of Fe^3+^ ions in the electrolyte. For higher Fe content materials (FeHf-BH-30.6, FeHf-BH-19, FeHf-BH-11.9, and FeHf-BH-8.2), the total charge involved in the PED experiments varies between 22.86 to 25.13 Q cm^–2^. These results may indicate the role of Hf or Fe contents on the quantity of the charge passed during PED deposition.

Moreover, to understand the effects of PED parameters on the electrode mass loading after deposition of FeHf-BH nanostructures, the *t*_on_ and total time were varied while keeping *V*_on_, *V*_off_, and *t*_off_ constant at −1.8 V, −0.5 V, and 1.0 s, respectively. Figure S4 shows the effect of varying *t*_on_ on the electrode mass loading after the deposition of FeHf-BH-30.6. It is evident that, for a constant total time (30 min), the mass loading increases to ∼22 mg cm^–2^ with an increase in *t*_on_ up to 0.5 s. Similarly, for a constant *t*_on_ (0.35 s), the electrode mass loading increases with an increase in the total PED time. These results clearly indicate the scalability of the procedure for depositing FeHf-BH materials.

**Figure 5:**
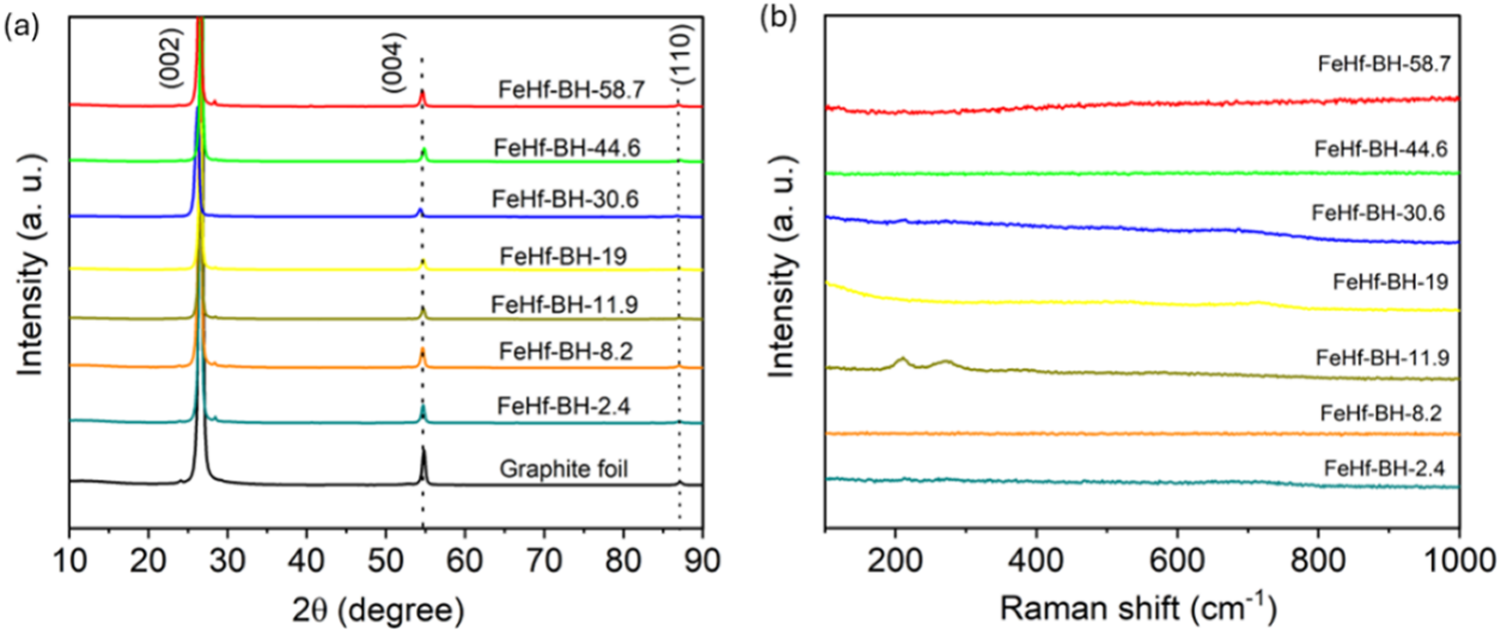
(a) XRD patterns and (b) Raman spectra of the FeHf-BH materials containing various Hf contents.

Figure 5a shows the XRD patterns for the as synthesized FeHf-BH materials containing various Hf contents and substrate (graphite foil). The graphite foil exhibited characteristic X-ray diffraction peaks at 2θ = 26.4, 55.1, and 87.4° indexing the (002), (004), and (110) planes respectively, corresponding to natural graphite with JCPDS no. 08-0415.^25^ All of the electrodeposited FeHf-BH nanostructures showed no signs of a diffraction pattern other than that of the graphite substrate, indicating the amorphous nature of the deposited material. These observations are consistent with previous studies on electrodeposited transition metal-based hydroxides/oxides from nitrate-based solutions.^18−22^ The micro Raman spectroscopy measurements were performed to investigate the nature of chemical bonding in the deposited materials. Figure 5b shows the Raman spectra of all of the FeHf-BH materials containing various Hf contents. The Raman spectra were mostly featureless for all of the FeHf-BH materials, suggesting the absence of crystalline phases in the material. From the XRD and Raman results, it turned out that all of the FeHf-BH materials are amorphous in nature. In order to understand the effect of heat treatment on the crystallinity of the electrodeposited FeHf-BH materials, the samples were treated at 500 °C for 2 h in an air atmosphere. The XRD results in Figure S5 indicated improved crystallinity for the annealed materials with the formation of thermodynamically stable oxide phases such as Fe_3_O_4_ and HfO_2_. For example, the thermally treated FeHf-BH-2.4 (FeHf-BH-2.4-T) showed peaks at 2θ = 28.3, 32.8, 58.6, 62.5, and 64.2° which may corresponds to (−111), (111), (−131), (131), and (113) planes of the monoclinic phase of HfO_2_ (JCPDS no. 06-0318).^26^ The same sample also showed peaks at 2θ of 35.4, 40.5, 50.0, and 66.5° which may corresponds to (221), (311), (400), and (511) planes of Fe_3_O_4_ (JCPDS no. 01–088–0315).^27^ Almost similar observations with the appearance of diffraction patterns from HfO_2_ and Fe_3_O_4_ were also noted in other thermally treated FeHf-BH materials (Figure S5, Supporting Information). The Raman modes for thermally treated FeHf-BH materials are presented in Figure S6 (Supporting Information). Most of the thermally treated FeHf-BH materials demonstrated intense Raman bands predominantly below 1000 cm^–1^.^28−31^ For example, the thermally treated FeHf-BH-11.9 (FeHf-BH-11.9-T) showed bands at 296 and 664 cm^–1^ which may correspond to A_1g_ and T_1g_ vibrational modes of Fe_3_O_4_,^28^ and the bands at 226, 401, and 604 cm^–1^ may be attributed to Fe_2_O_3_.^28,29^ Moreover, the sample also showed an additional band at 499 cm^–1^ from A_g_ Raman mode of monoclinic HfO_2_.^31^ Similarly, most of the thermally threated materials, e.g., FeHf-BH-2.4-T, FeHf-BH-19-T, FeHf-BH-30.6-T, and FeHf-BH-44.6-T showed chemical transformation from amorphous oxide phases to crystalline phases of Fe_3_O_4_, Fe_2_O_3_, and HfO_2_ as the result of thermal oxidation. However, the thermally treated materials did not adhere well to the graphite electrode surface and may require additional steps or processing methods to fix them on the electrode surface. Therefore, the annealed samples were not examined in terms of catalytic activity toward the OER reaction, and catalytic tests were carried out only on the materials as received after electrodeposition.

**Figure 6:**
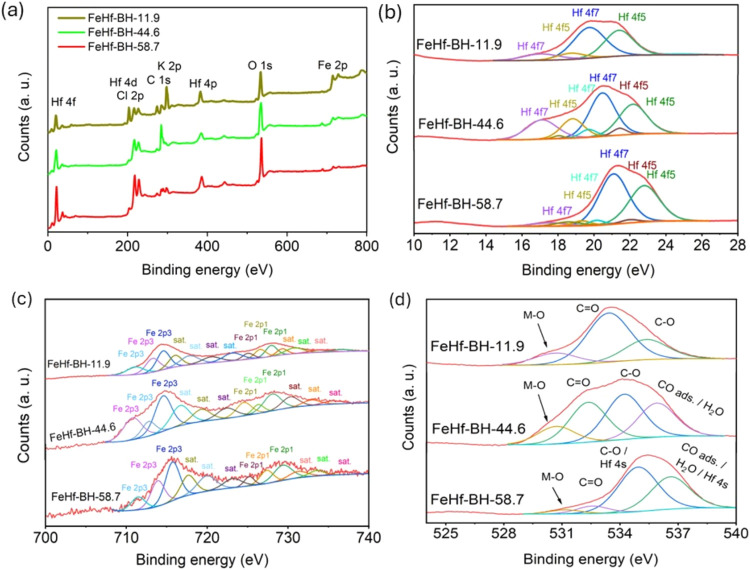
(a) XPS survey spectra of FeHf-BH-11.9, FeHf-BH-44.6, and FeHf-BH-58.7 nanostructures. The high-resolution XPS spectra of (b) Hf 4f, (c) Fe 2p, and (d) O 1s for FeHf-BH-11.9, FeHf-BH-44.6, and FeHf-BH-58.7 materials.

Further, XPS analyses were conducted to investigate the bonding environment and oxidation states of Fe and Hf in the electrodeposited materials. Figure 6a shows the XPS survey spectra of FeHf-BH-11.9, FeHf-BH-44.6, and FeHf-BH-58.7. All of the materials showed the presence of Fe, Hf, O, C, and trace impurities (Cl and K) from electrolytes. The deconvoluted high-resolution Hf 4f spectra of all of the materials were presented and compared in Figure 6b. The Hf 4f_7/2_ peaks at 17.1 eV for FeHf-BH-11.9 and FeHf-BH-44.6 and the same Hf 4f_7/2_ peak at 17.6 eV for FeHf-BH-58.7 may corresponds to Hf–O bonding in HfO_2_.^4,32,33^ The positive shift of the Hf 4f_7/2_ peak toward higher binding energies for the FeHf-BH-58.7 sample may indicate the effect of increasing Hf/Fe ratio on the electronic structure of Hf. Figure 6c shows the deconvoluted high-resolution Fe 2p spectra for FeHf-BH-11.9, FeHf-BH-44.6, and FeHf-BH-58.7. The Fe 2p spectra of all of the samples were appeared to be similar to the presence of Fe 2p_3/2_ and Fe 2p_1/2_ spin–orbit peaks and some corresponding shakeup satellite peaks. Moreover, for all of the samples, the data fitting of Fe 2p_3/2_ in the range of 707–717 eV showed three peaks. The Fe 2p_3/2_ located at 711.1, 710.8, and 711.5 eV for FeHf-BH-11.9, FeHf-BH-44.6, and FeHf-BH-58.7, respectively, suggests the presence of Fe–O bonding, likely from iron(III) oxide/hydroxide phases.^34,35^ Furthermore, the higher binding energy for Fe 2p_3/2_ in the FeHf-BH-58.7 samples compared to those in the other samples with low Hf content may indicate an effective electronic interaction and charge transfer processes between Fe and Hf, which in turn can influence the OER performance of composites. Similar observations with shifting of the Co 2p_3/2_ peak in case of the Hf-modified CoOOH/Co(OH)_2_ system were also reported by R. Zhu et al.^4^ Further, the deconvoluted high-resolution O 1s spectrum for the samples is presented in Figure 6d. For FeHf-BH-11.9, the deconvoluted O 1s spectra at 530.7, 533.4, and 535.3 eV can be attributed to M-O bonding (where M: Hf and Fe), C=O, and C–O respectively.^36,37^ Similarly, the O 1s spectrum for FeHf-BH-44.6 and FeHf-BH-58.7 can be deconvoluted into four components centered at 530.7 eV (M-O), 532.4 eV (C=O), 534.2 eV (C–O), and 535.9 eV (CO adsorbed/H_2_O). The presence of C–O and C=O structures in the materials indicates surface oxidation of the graphite substrate.^36,37^ Hence, the above analyses clearly indicate electrodeposition of hydroxides/oxides comprising Fe(III) and Hf(IV).

**Figure 7:**
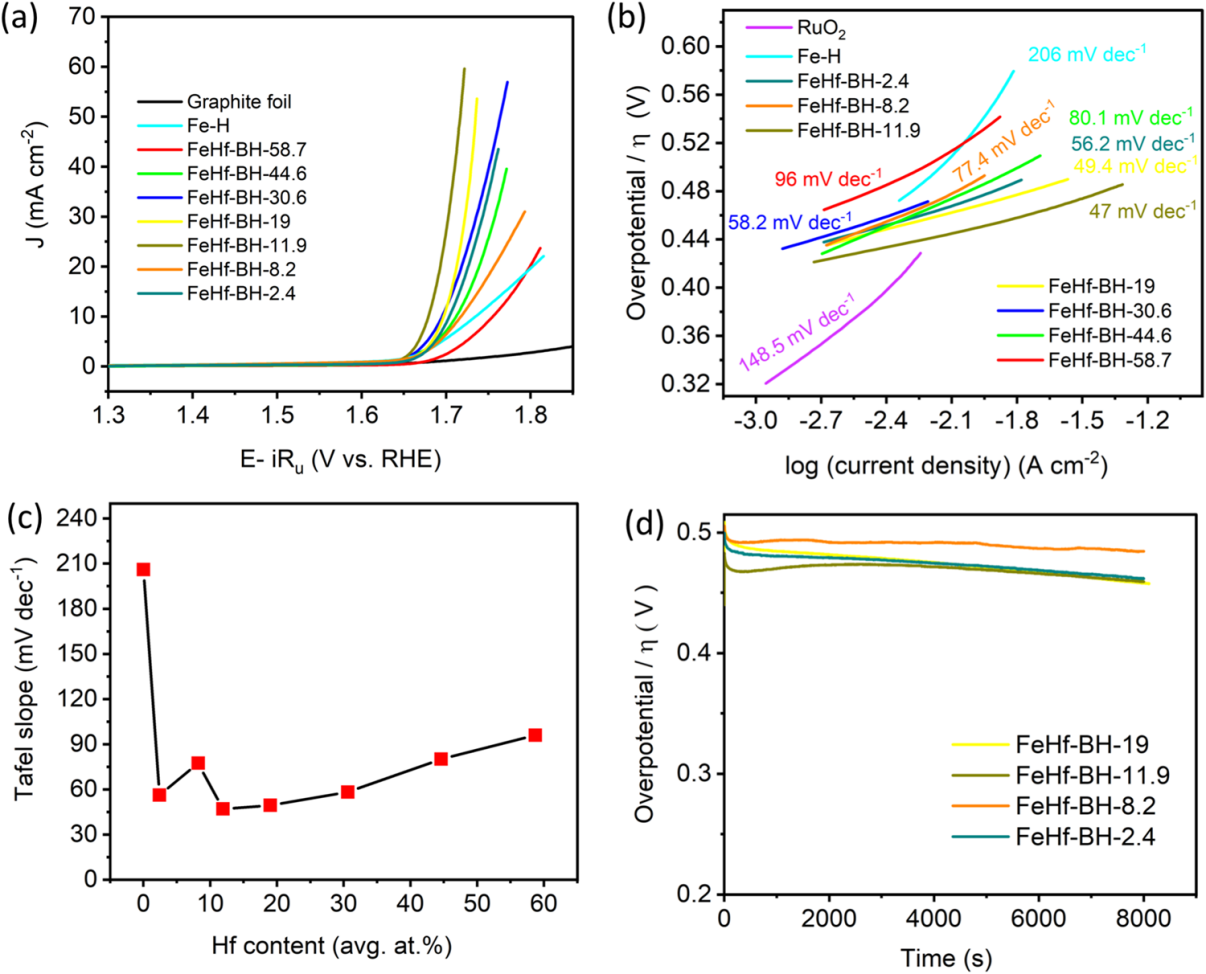
(a) LSV curves of OER measurements for the electrodeposited FeHf-BH materials, graphite foil, and Fe–H in 1 M KOH. (b) Corresponding Tafel plots for the materials along with a commercial RuO_2_ electrocatalyst. (c) Variation of OER Tafel slope values with respect to the Hf content. (d) OER chronopotentiometry measurements at 10 mA cm^–2^ for FeHf-BH-19, FeHf-BH-11.9, FeHf-BH-8.2, and FeHf-BH-2.4 electrodes.

Previously, in most of Fe-containing multimetal-based hydroxide/oxide systems (e.g., FeW oxide, Fe–Ta_2_O_5_, Co-FeO_*x*_, Fe–Co_3_O_4_, Au@CoFeO_*x*_, Ni–Fe–Co mixed oxide, Ni–Fe oxide, Co–Fe oxide, FeCoOOH/G, and NiFe LDH/CNT), Fe(II or III) is considered the active center for the oxygen evolution reaction (OER) in an alkaline medium.^38−47^ Therefore, in the present case, the OER activity of FeHf-BH materials (Figure 7a) was evaluated in an alkaline medium (1 M KOH). For comparison, the OER activities of substrate (graphite foil), commercial RuO_2_ catalyst, and Fe-hydroxide (Fe–H) without Hf dopants were evaluated under similar conditions. The linear sweep voltammetry (LSV) results for the OER in Figure 7a depict a significant role of the Hf content in the OER activity of FeHf-BH materials. The hydroxide material without the Hf content required 1.73 V to attain an OER current density of 10 mA cm^–2^, which is more positive compared with most of the Hf-containing FeHf-BH materials. The lowest activity, in terms of the Hf content, was exhibited by the highest-activity Hf-containing material (FeHf-BH-58.7), which required 1.75 V to reach 10 mA cm^–2^. However, with a decrease in the Hf content compared to FeHf-BH-58.7, the OER activity of the materials increased significantly. Both FeHf-BH-30.6 and FeHf-BH-19 required 1.69 V, while FeHf-BH-44.6 required 1.71 V to achieve an OER current density of 10 mA cm^–2^. With further decrease in the Hf content, FeHf-BH-11.9 showed 1.67 V to attend the same current density. However, with an additional decrease in the Hf content, the OER activity declined again. The FeHf-BH-8.2 and FeHf-BH-2.4 required 1.71 and 1.70 V, respectively, to reach a 10 mA cm^–2^ OER current. Moreover, the comparison of the OER LSV curves with a commercial RuO_2_ electrocatalyst and FeHf-BH-11.9 shown in Figure S7 clearly demonstrates the higher activity of FeHf-BH-11.9 in terms of the OER current. Although the commercial RuO_2_ catalyst exhibited a more negative OER onset potential (1.55 V) compared to that of FeHf-BH-11.9 (1.63 V), it required 1.71 V to reach the OER current of 10 mA cm^–2^, which is 40 mV more positive than 1.67 V required by FeHf-BH-11.9. These results clearly highlight the role of Hf content in the OER activity of the studied materials, indicating that an optimized Hf:Fe ratio or the Hf content is beneficial for OER in an alkaline medium.

**Figure 8:**
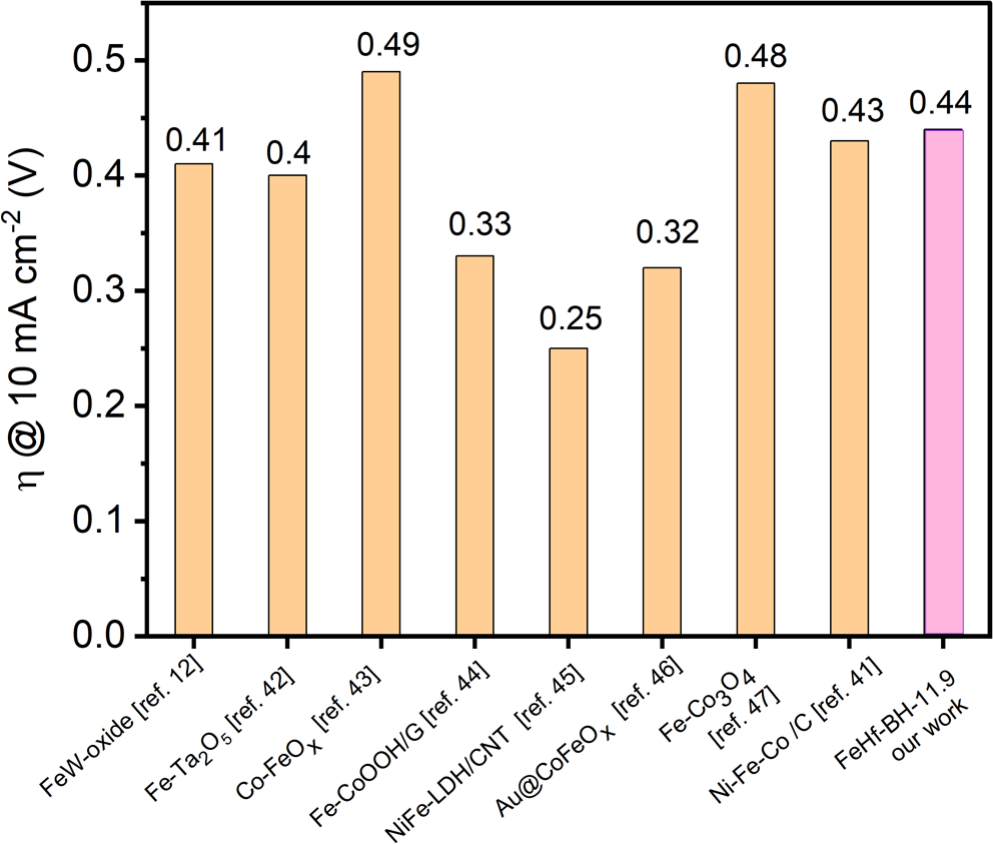
Comparison of the OER overpotential (η) at the 10 mA cm^–2^ OER current density for FeHf-BH-11.9 with other reported Fe-containing materials.

Tafel analyses were performed to gain insight into the kinetics of the OER in these systems. The Tafel plots for all of the FeHf-BH materials, along with RuO_2_ and Fe–H, were derived from their respective LSV curves (Figure 7b). All of the FeHf-BH materials showed lower Tafel slope values compared to RuO_2_ (148.5 mV dec^–1^) and Fe–H (206 mV dec^–1^). Figure 7c shows the Tafel slope values as a function of the Hf content. The Tafel slope values decreased from 96 mV dec^–1^ (FeHf-BH-58.7) to 47 mV dec^–1^ (FeHf-BH-11.9) with a decrease in the Hf content, before increasing to 77.4 mV dec^–1^ (FeHf-BH-8.2) with further decreases in the Hf content. Therefore, the lowest Tafel slope for FeHf-BH-11.9 indicates rapid kinetics of the OER process. Nevertheless, these results further suggest the role of optimization of Hf contents for enhanced OER activity. The catalytic stability of the FeHf-BH materials was tested by chronopotentiometry at an OER current density of 10 mA cm^–2^ (Figure 7d). All four catalysts under investigation, with a low Hf content (below 20 at. %), exhibited high stability for up to 8000 s, with very minimal fluctuations in overpotentials. These results indicate that the FeHf-BH materials are stable under the OER conditions and may be suitable for long-term operation. Figure 8 shows the comparison of the OER activity of FeHf-BH-11.9 with some of the reported Fe-containing multimetal-based oxide/hydroxide composites. As can be seen, the observed OER overpotential is close to values previously reported in the literature for other iron-based materials.

## 4 Conclusions

In summary, we demonstrated the coelectrodeposition of FeHf binary hydroxides/oxides from NO_3_^–^ ions containing electrolytes. A systematic study was conducted to investigate the effects of electrolyte composition (Fe^3+^/Hf^4+^ ratio) and PED parameters on the morphology, stoichiometry, and crystallinity of the deposited materials. By controlling the Fe^3+^/Hf^4+^ ratio in the electrolyte, we achieved a controlled variation in Hf (2.4–58.7 avg. at. %) and Fe (5.9–49.9 avg. at. %) content in the deposited materials. Additionally, we showed the ability to control the loading mass of FeHf-BH electrodes (∼4 to 24 mg cm^–2^) by tuning the PED parameters, specifically *t*_on_ and total deposition time. The structural morphology of deposits observed through FE-SEM revealed agglomerated nanoparticles with sizes ranging from 50 to 150 nm. XRD and Raman studies indicated improved crystallinity in the thermally annealed materials compared with freshly prepared ones, with crystalline phases corresponding to Fe_3_O_4_, Fe_2_O_3_, and HfO_2_. XPS analyses confirmed the presence of Fe in the 3+ oxidation state and Hf in the 4+ state in the deposited materials. The effect of varying Hf to Fe ratio in the obtained materials on the oxygen evolution reaction was investigated in an alkaline medium. The material with the optimized Hf:Fe ratio (FeHf-BH-11.9) required a lower overpotential for the OER compared to other ratios and a commercial RuO_2_ electrocatalyst. Notably, a variation in Tafel slope values were observed depending on the Hf content in the samples, with the lowest Tafel slope (47 mV dec^–1^) achieved for FeHf-BH-11.9, indicating enhanced OER kinetics in an alkaline medium. This work highlights the scalability and tunability of the PED process to deposit Fe and Hf binary hydroxides/oxides from nitrate-based electrolytes and demonstrates the critical role of the Fe/Hf ratio in enhancing the intrinsic electrocatalytic activity of these materials. It is worth noting that this strategy may inspire the design of new transition-metal-based hydroxides/oxides for electrocatalytic applications.

## Data Availability

The data provided in this study are available at RODBUK Cracow Open Research Data Repository at https://doi.org/10.57903/UJ/YQ2LMP.

## Supporting Information Available

Materials synthesis; digital photographs; EDS analysis results; graph for total charge vs Hf and Fe contents; graph for *t*_on_ vs electrode mass loading; graph for total time (*T*) vs electrode mass loading; XRD spectra; Raman spectra; LSV curves (PDF)
