# Supplementary material for: FeHf Binary Hydroxide/Oxide Nanostructures as Catalysts for Oxygen Evolution
Source: ACS Appl Nano Mater. 2025 Apr 19;8(17):8865–75. doi: 10.1021/acsanm.5c00912 (PMC12053830; doi:10.1021/acsanm.5c00912)
Supplement: Supplementary file 1 — an5c00912_si_001.pdf [file an5c00912_si_001.pdf]

Supporting Information

FeHf Binary Hydroxide/Oxide Nanostructures as Catalysts for Oxygen Evolution

Biswaranjan D. Mohapatra[1*], Mateusz Szczerba[1,2], Joanna Czopor[1], Daniel Piecha[1,2], Marcin Pisarek[3], Grzegorz D. Sulka[1*]

[1]Department of Physical Chemistry and Electrochemistry, Faculty of Chemistry, Jagiellonian University, Gronostajowa 2, 30-387, Krakow, Poland

[2]Doctoral School of Exact and Natural Sciences, Jagiellonian University, Lojasiewicza 11, 30-348 Krakow, Poland

[3]Laboratory of Surface Analysis, Institute of Physical Chemistry, Polish Academy of Sciences, Kasprzaka 44/52, 01-224 Warsaw, Poland

Corresponding authors' e-mail addresses: biswaranjan.research@gmail.com, sulka@chemia.uj.edu.pl

Table S1. The concentration of $FeCl_3$ and $HfCl_4$ and their molar ratios in the electrolytes.

Conc. of $FeCl_3$ (mM)	Conc. of $HfCl_4$ (mM)	Molar ratio of Fe^{3+}: Hf^{4+}
90	10	9:1
90	30	3:1
90	50	9:5
50	50	1:1
15	50	3:10
5	50	1:10

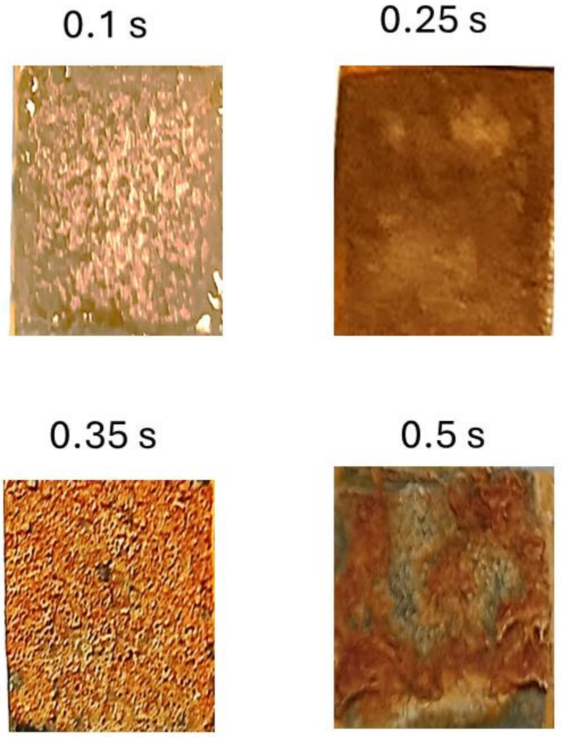

Figure S1. The photographic images of the deposited materials with different t_{on} durations, i.e., 0.1, 0.25, 0.35, and 0.5 s from the electrolyte containing 30 and 50 mM of Fe^{3+} and Hf^{4+} ions.

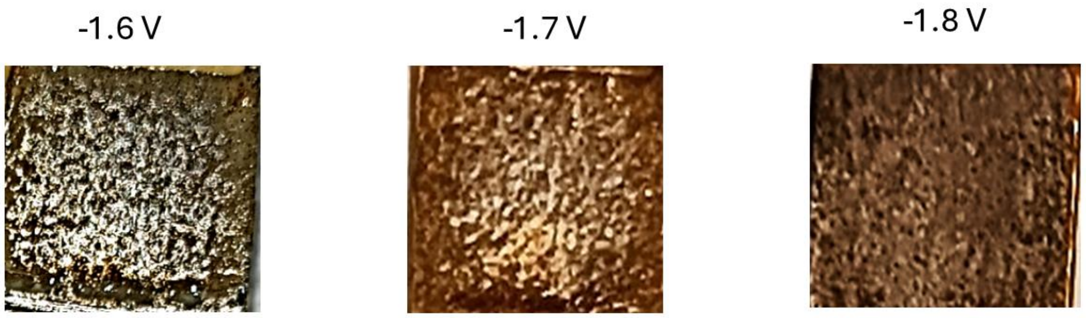

Figure S2. The photographic images of the deposited materials with different potentials of V_{on} from the electrolyte containing 30 and 50 mM of Fe^{3+} and Hf^{4+} ions.

Table S2. The average at.% of Fe and Hf in the deposited FeHf-BH materials obtained by analyzing the EDS spectra at five arbitrary points under FE-SEM.

Molar ratio of Fe^{3+}:Hf^{4+} in the electrolyte	Average at.% of Fe	Standard deviation for Fe (at.%)	Average at.% of Hf	Standard deviation for Hf (at.%)
9:1	49.9	2.7	2.4	0.5
3:1	34.9	5.0	8.2	2.1
9:5	35.6	7.1	11.9	0.7
1:1	27.0	2.2	19.0	1.7
3:5	32.5	2.5	30.6	3.6
3:10	11.8	1.7	44.6	2.0
1:10	5.9	0.5	58.7	0.8

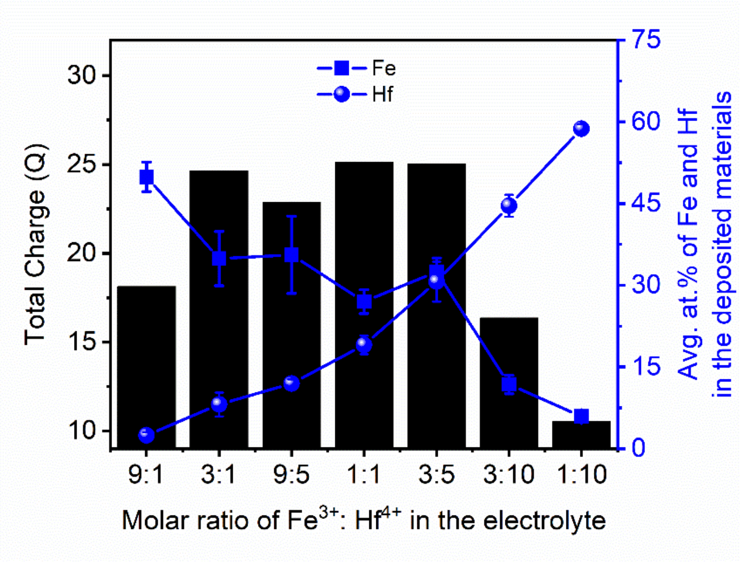

Figure S3. The total charge involved, and average at.% of Fe and Hf for the deposited materials obtained at PED conditions of -1.8 V (V_{on}), 0.35 s (t_{on}), -0.5 V (V_{off}), and 1.0 s (t_{off}) for 30 min.

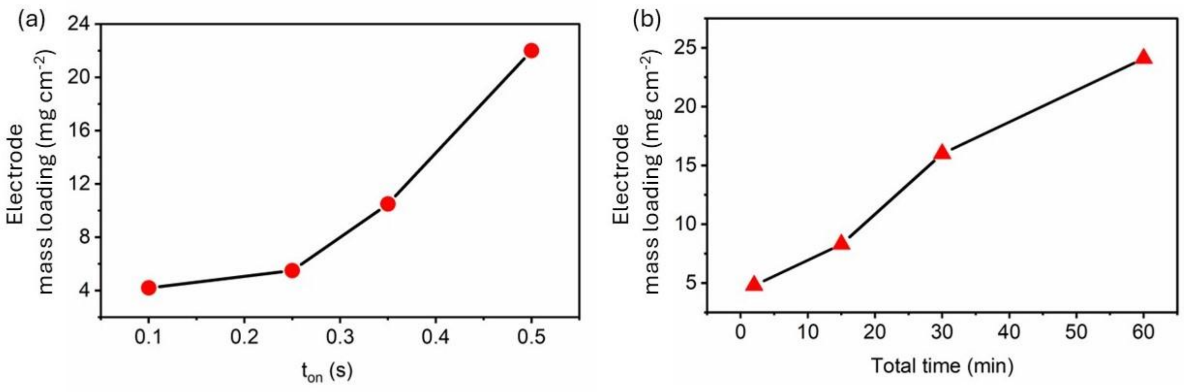

Figure S4. (a) Effect of varying t_{on} on the electrode mass loading after deposition of FeHf-BH-30.6 (V_{on} = -1.8 V, V_{off}= -0.5 V, t_{off} = 1 s, total time = 30 min). (b) Effect of varying total time on the electrode mass loading after deposition of FeHf-BH-8.2 (V_{on} = -1.8 V, t_{on} = 3.5 s, V_{off}= -0.5 V, t_{off} = 1 s).

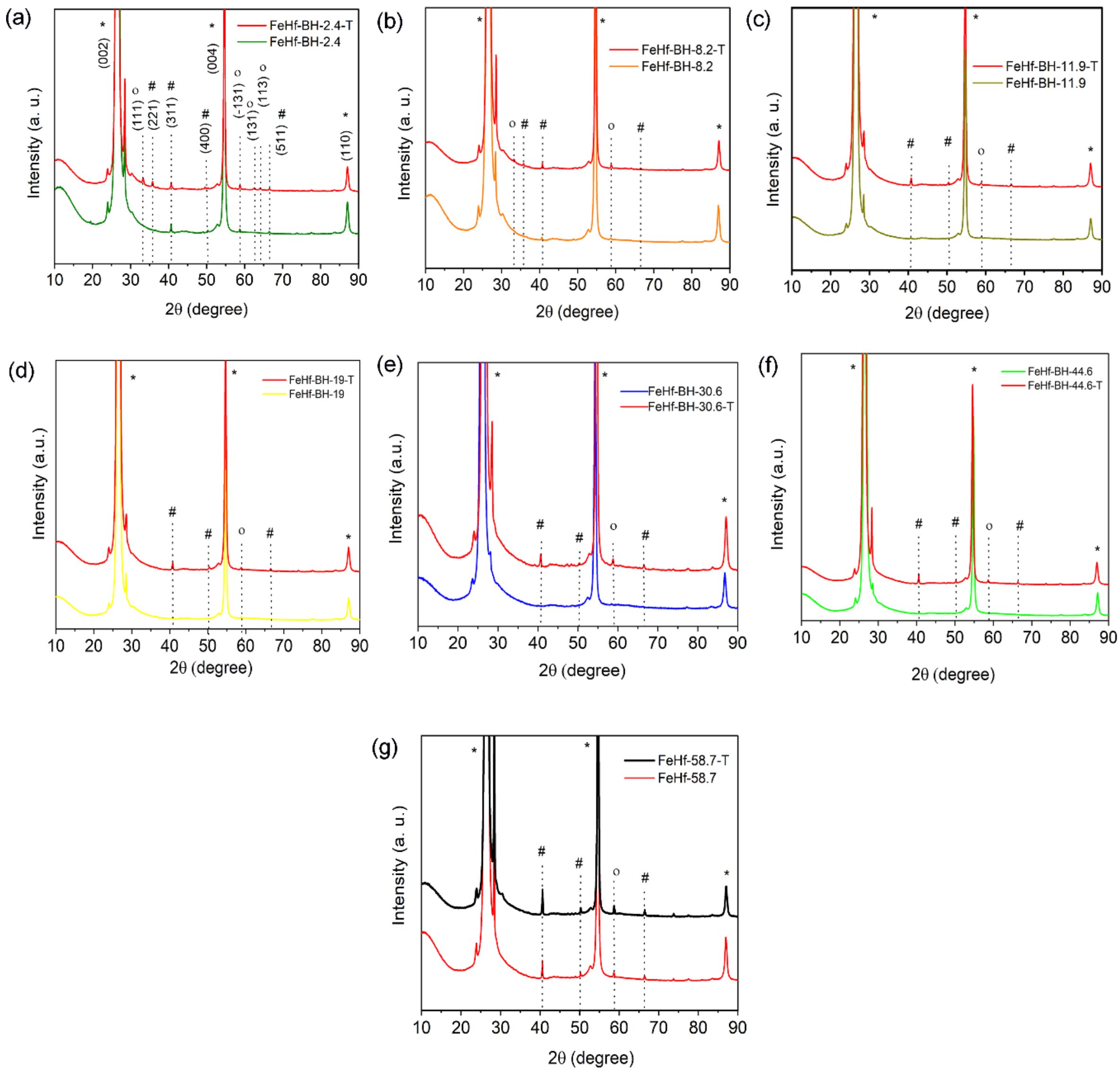

Figure S5. XRD patterns comparisons for (a) FeHf-BH-2.4 and FeHf-BH-2.4-T, (b) FeHf-BH-8.2 and FeHf-BH-8.2-T, (c) FeHf-BH-11.9 and FeHf-BH-11.9-T, (d) FeHf-BH-19 and FeHf-BH-19-T, (e) FeHf-BH-30.6 and FeHf-BH-30.6-T, (c) FeHf-BH-44.6 and FeHf-BH-44.6-T and (g) FeHf-BH-58.7 and FeHf-BH-58.7-T. The *, # and o marks in the graphs represents the lattice planes from graphite, Fe_3O_4, and HfO_2 respectively.

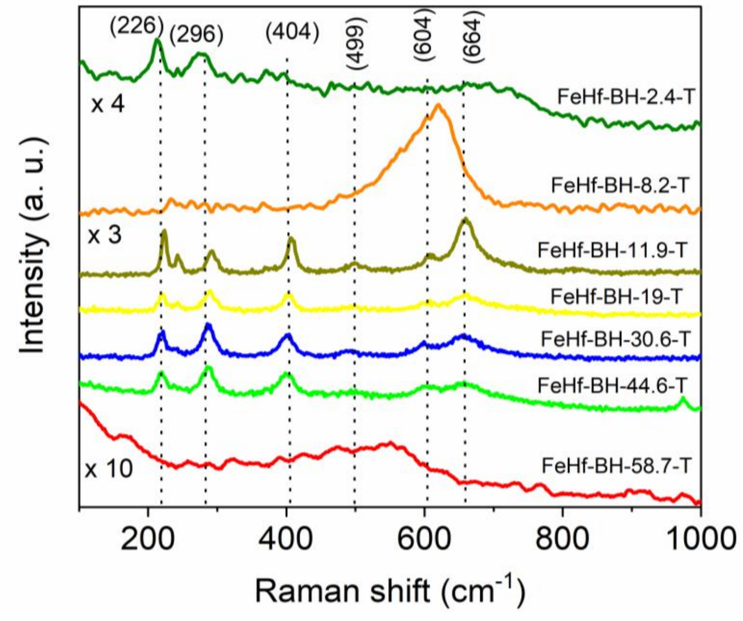

Figure S6. Raman spectra of FeHf-BH-2.4-T, FeHf-BH-8.2-T, FeHf-BH-11.9-T, FeHf-BH-19-T, FeHf-BH-30.6-T, FeHf-BH-44.6-T, and FeHf-BH-58.7-T.

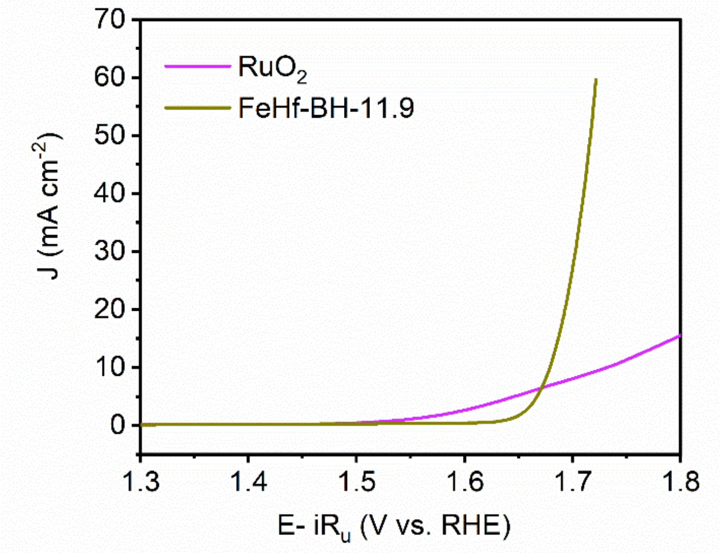

Figure S7. LSV curves of OER measurements for the electrodeposited FeHf-BH-11.9 sample and commercial RuO_2 in 1.0 M KOH.
